# Role of paternal *Oryza sativa Baby Booms* (*OsBBMs*) in initiating *de novo* gene expression and regulating early zygotic development in rice

**DOI:** 10.1111/tpj.70305

**Published:** 2025-06-26

**Authors:** Nargis Akter, Takumi Tezuka, Kasidit Rattanawong, Aya Satoh, Atsuko Kinoshita, Yutaka Sato, Takashi Okamoto

**Affiliations:** ^1^ Department of Biological Sciences Tokyo Metropolitan University Tokyo 192‐0397 Japan; ^2^ Department of Food Technology and Nutritional Science Mawlana Bhashani Science and Technology University Tangail 1902 Bangladesh; ^3^ National Institute of Genetics Mishima Shizuoka 411‐8540 Japan

**Keywords:** OsBBMs, paternal allele, zygote, development, fertilization, transcriptome analysis, AP2 transcription factor, *Oryza sativa*

## Abstract

*Oryza sativa* BABY BOOM 1 (OsBBM1), a member of the AP2/ERF family of transcription factors, is expressed from paternal allele in rice zygote and plays a crucial role in initiating zygotic development. However, the mechanism how the paternal OsBBM1 drives this development remains unclear. Rice zygotes with four different gamete combinations with or without functional paternal OsBBMs were produced by electrofusion, using gametes isolated from *bbms* triple mutants and wild‐type rice plants. Developmental and gene expression profiles of these types of zygotes were intensively analyzed and compared. Mutations in *OsBBM1*, *OsBBM2*, and *OsBBM3* on the paternal alleles caused developmental arrest or delay in the zygotes, while defects in *OsBBMs* on the maternal allele had minimal effects on zygotic development. Paternal allele of *OsBBMs* significantly influenced gene expression profiles related to regulation of basic cellular processes, such as chromosome/chromatin organization/assembly and cell cycle/division compared to the maternal allele of *OsBBMs*. Majority of these genes were upregulated in zygotes from paternal/parental alleles via paternal *OsBBMs*. Paternal *OsBBMs* initiate early development of rice zygotes through the regulation of expression profiles of genes controlling status of chromosome/chromatin and cell cycle/division.

## Significance Statement

*Oryza sativa* BABY BOOM 1 (OsBBM1) belonging to the AP2/ERF family transcription factor plays an important role in initiating developmental programs in rice zygotes. In this study, we demonstrate that paternal *OsBBMs* contribute to establish the cellular frameworks necessary for proper zygotic development through the regulation of expression profiles on genes possibly related to chromosome/chromatin organization/assembly, reorganization of cellular polarity, and cell cycle/division, and cell wall formation.

## INTRODUCTION

Upon the fusion of a sperm cell with an egg cell in angiosperms, karyogamy (nuclear fusion) between the male and female nuclei rapidly progresses in the zygote (Ohnishi et al., 2014), and the combined parental genomes synergistically function to drive early embryogenesis (Toda et al., 2018; Zhao et al., 2017). To investigate the mechanisms underlying this synergy, genes specifically or preferentially expressed from paternal or maternal alleles have been identified in zygotes and/or early embryos of Arabidopsis (Gehring et al., 2011; Hsieh et al., 2011; Raissig et al., 2013), maize (Jahnke & Scholten, 2009; Waters et al., 2011), and rice (*Oryza sativa*; Luo et al., 2011). Among these genes, *O. sativa Apospory‐specific Genome Region* (*ASGR*)‐*BABY‐BOOM LIKE* (*BBML*) *1*, termed *OsASGR‐BBML1* or *OsBBM1*, is preferentially expressed from the paternal allele in rice zygotes and encodes an AP2‐type transcription factor that initiates zygotic development (Khanday et al., 2019; Rahman et al., 2019).

The *ASGR‐BBML* gene was first identified in the ASGR of *Pennisetum squamulatum*, which controls apomeiosis and parthenogenesis (Ozias‐Akins et al., 1998). PsASGR‐BBML1 was later shown to induce parthenogenesis in the egg cells of sexual pearl millet (Conner et al., 2015). Additionally, the ectopic expression of *PsASGR*‐*BBML1* in maize and rice egg cells induces haploid embryo production (Conner et al., 2017). In rice, *OsASGR‐BBML1* (Os11g0295900, alternative gene name *OsBBM1*) is an ortholog of *PsASGR‐BBML1*, with three homologous genes—*OsBBM2*, *OsBBM3*, and *OsBBM4*—identified. Of these four genes, *OsBBM1*, *OsBBM2*, and *OsBBM3* are known to be expressed in zygotes (Khanday et al., 2019; Rahman et al., 2019).

OsBBM1 has been proposed as a key initiator of zygotic development in rice, as the ectopic expression of *OsBBM1* induces autonomous egg cell division (Rahman et al., 2019), and when combined with mutations affecting meiosis, *OsBBM1* ectopic expression results in the formation of clonal progeny (Khanday et al., 2019). These findings suggest that exogenous *OsBBM1* can shift egg cells from a quiescent to a proliferative state. However, *in planta*, autonomous egg cell development without fusion with sperm cells is suppressed, as the expression of *OsBBM1* and its homologs is strictly suppressed in egg cells and *OsBBM1* in zygotes is initially derived only from the paternal side (genome) after fusion with sperm cells. This synergistic effect in parental genomes on *OsBBM1* expression will be essential for avoiding autonomous cell division/proliferation without gamete fusion. It can be hypothesized that monoallelic or preferential gene expression from the paternal genome in the zygote is a safety mechanism for the egg cell, allowing it to suppress the gene expression cascade toward embryogenesis, which is normally triggered by fusion with a sperm cell, and only the paternal allele is initially active in the zygote after fertilization (Rahman et al., 2019). This monoallelic expression likely prevents autonomous development without sperm fusion, safeguarding against untimely embryogenesis. Therefore, understanding how *OsBBM1* regulates gene expression in zygotes is essential to uncovering the mechanisms of early embryonic development.

It has been suggested that paternal *OsBBM1* activates the *OsBBM1* maternal allele in rice zygotes (Khanday et al., 2019). Further research by Khanday et al. (2023) demonstrated that *OsBBM1* directly upregulates *OsYUCCA* auxin biosynthesis genes preferentially from the maternal genome in callus tissues, implying that the paternal *OsBBM1*–maternal *OsYUC* module may be a possible key player for zygotic development. Despite these findings, the molecular mechanisms by which *OsBBM1* initiates development remain unclear, as they likely involve the regulation of basic cellular processes, such as the cell cycle, chromosome organization, and cell polarity.

In this study, to clarify the molecular mechanisms of OsBBM‐dependent development of rice zygotes and early embryos, we analyzed the effect of the loss of function of *OsBBMs* in zygotes by using a homozygous nucleotide insertion triple *bbm1bbm2bbm3* mutant line (*bbms*) generated by the CRISPR/Cas9 genome editing system. Using the rice *in vitro* fertilization (IVF) system (Okamoto, 2010; Uchiumi et al., 2007), gametes isolated from *bbms* triple mutant rice plants were fused to create *bbms*‐mutant zygotes with defects in both and either paternal or maternal alleles (*bbms* egg‐*bbms* sperm, *bbms* egg‐WT sperm, and WT egg‐*bbms* sperm), and the developmental profiles of these zygotes were monitored. We detected a significant delay in development in the zygotes possessing *bbms* mutations on the paternal allele but not in zygotes harboring *bbms* mutations on the maternal allele. To precisely compare the early developmental state of zygotes possessing *bbms* mutations in the paternal or maternal allele, changes in nuclear (cell) numbers of early embryos during the development of zygotes with three gametes combination (WT egg‐WT sperm, *bbms* egg‐WT sperm, and WT egg‐*bbms* sperm) was counted. Furthermore, we performed transcriptome analysis of rice zygotes/embryos with four combinations (WT egg‐WT sperm, *bbms* egg‐*bbms* sperm, *bbms* egg‐WT sperm, and WT egg‐*bbms* sperm) at four early developmental stages (4, 18, 42, and 66 h post‐fusion). Through weighted gene co‐expression network analysis (WGCNA) and single nucleotide polymorphism (SNP)‐based transcriptome analyses, we identified genes influenced by paternal *OsBBM* alleles. This study provides new insight into the role of OsBBM in initiating zygotic development in rice.

## RESULTS

### Impact of paternal and maternal *OsBBM* alleles on zygotic development

In this study, genome‐edited rice plants, termed *bbms*, where homozygous knockout mutations were introduced into *OsBBM1*, *OsBBM2*, and *OsBBM3* via genome editing (Figure S1), were used for zygote production. It has been suggested that these three *BBM* genes are expressed in rice zygotes and function redundantly (Khanday et al., 2019; Rahman et al., 2019). Using gametes isolated from both wild‐type (WT) and *bbms* triple mutant rice plants, zygotes with four gamete combinations—WT egg‐WT sperm, *bbms* egg‐*bbms* sperm, *bbms* egg‐WT sperm, and WT egg‐*bbms* sperm—were produced by an electro‐fusion‐mediated IVF system (Uchiumi et al., 2007). The resulting zygotes were cultured and their developmental profiles monitored and compared.

**Figure 1:**
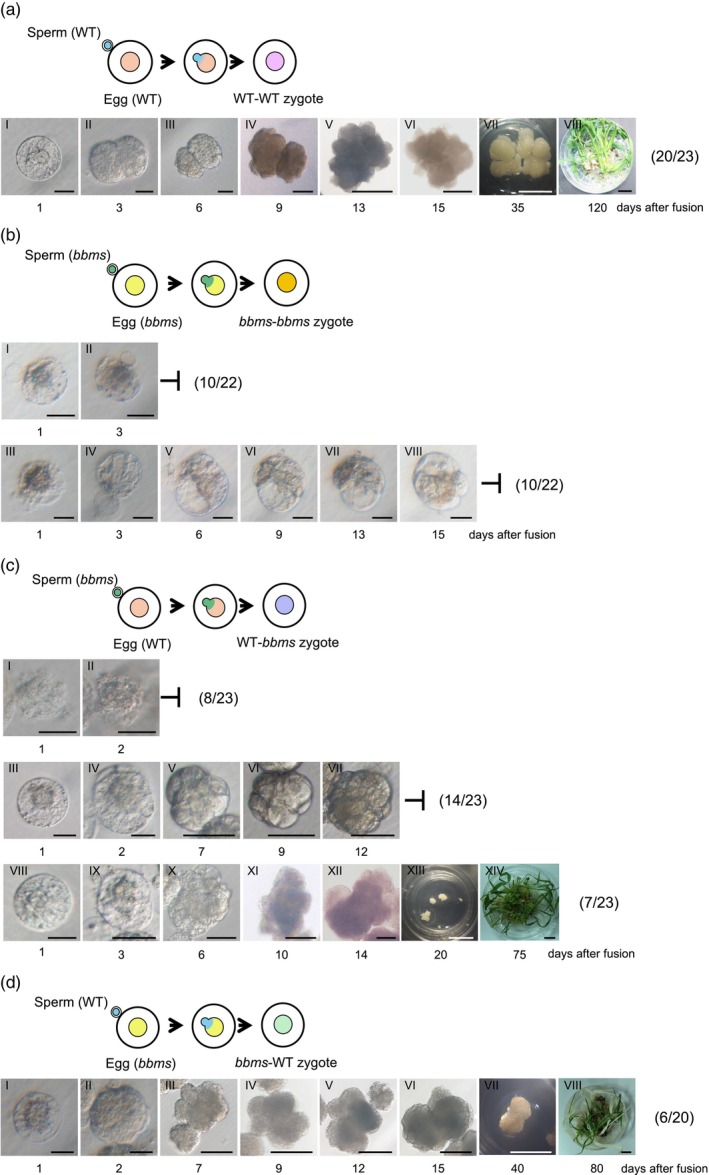
Effects of *OsBBMs* derived from paternal or maternal alleles on the developmental profiles of rice zygotes. (a) A WT‐WT zygote was produced by the fusion of gametes isolated from wild‐type (WT) rice plants (top panel), and the developmental profile of the resulting zygote was observed (bottom panel). A WT‐WT zygote developed into a two‐celled embryo at 1 day after fusion (Panel I), a multicellular structure at 3 days after fusion (Panel II), a globular‐like embryo at 6 days after fusion (Panel III), and a cell mass at 9–15 days after fusion (Panels IV–VI). The cell mass subsequently developed into a white callus (Panel VII), and regenerated into a plantlet (Panel VIII). The proportion 20/23 in parentheses represents the ratio between the number of developed zygotes that regenerated into a plantlet and the number of zygotes produced. (b) A *bbms‐bbms* zygote was produced by the fusion of gametes isolated from *bbms* triple mutant plants (top panel), and the developmental profiles of the resulting zygote were observed (middle and bottom panels). A *bbms‐bbms* zygote degenerated without division (Panels I–II). Alternatively, a *bbms‐bbms* zygote remained in the one‐celled stage until 3 days after fusion (Panels III–IV) and developed into a possible two‐celled embryo at 6 and 9 days after fusion (Panels V–VI), a multicellular structure at 13 days after fusion (Panel VII), and a globular‐like embryo at 15 days after fusion (Panel VIII). Thereafter, the globular embryo exhibited developmental arrest and degenerated. The proportion 10/22 in parentheses at middle panel represents the ratio between the number of degenerated zygotes and the number of zygotes produced. The proportion 10/22 in parentheses at bottom panel represents the ratio between the number of developed zygotes/embryos and the number of zygotes produced. (c) A WT*‐bbms* zygote was produced by the fusion of a WT egg cell and *bbms* sperm cell (first panel), and the developmental profiles of the resulting zygote were observed (second, third, and fourth panels). A WT‐*bbms* zygote degenerated without division (Panels I–II). Alternatively, a WT‐*bbms* zygote was detected in the one‐celled stage at 1 day after fusion (Panel III), developed into a possible two‐celled embryo at 2 days after fusion (Panel IV), and a globular‐like embryo at 7–12 days after fusion (Panels V–VII). The globular‐like embryo exhibited developmental arrest and subsequently degenerated. Another WT‐*bbms* zygote was detected in the one‐celled stage at 1 day after fusion (Panel VIII), developed into a possible two‐celled embryo at 3 days after fusion (Panel IX), a multicellular structure at 6 days after fusion (Panel X), and a cell mass at 10–14 days after fusion (Panels XI–XII). The cell mass developed into a white callus (Panel XIII) and regenerated into a plantlet (Panel XIV). The proportion 8/23 in parentheses at second panel represents the ratio between the number of degenerated zygotes and the number of zygotes produced. The proportion 14/23 in parentheses at third panel represents the ratio between the number of developed zygotes/embryos and the number of zygotes produced. The proportion 7/23 in parentheses at fourth panel represents the ratio between the number of developed zygotes that regenerated into a plantlet and the number of zygotes produced. (d) A *bbms*‐WT zygote was produced by the fusion of a *bbms* egg cell and WT sperm cell (top panel), and the developmental profile of the resulting zygote was observed (bottom panel). A *bbms*‐WT zygote developed into a possible two‐celled embryo at 1 days after fusion (Panel I), a globular‐like embryo at 2 days after fusion (Panel II), and a cell mass at 7–15 days after fusion (Panels III–VI). The cell mass developed into a white callus (Panel VII) and regenerated into a plantlet (Panel VIII). The proportion 6/20 in parentheses represents the ratio between the number of developed zygotes that regenerated into a plantlet and the number of zygotes produced. Color‐coding for gamete and zygote genotypes: (a) orange, light blue, and pink circles indicate the nuclei/chromatins in the WT egg cell, WT sperm cell, and WT‐WT zygote, respectively. (b) Yellow, green, and bronze circles indicate the nuclei/chromatins in the *bbms* egg cell, *bbms* sperm cell, and *bbms*‐*bbms* zygote, respectively. (c, d) Purple and olive circles indicate the nuclei/chromatins in the WT‐*bbms* and *bbms*‐WT zygote, respectively. Scale bars: 20 μm (a, Panels I–III; b, Panels I–VIII; c, Panels I–IV; c, Panels VIII–X; d, Panels I–II); 50 μm (a, Panel IV; b, Panels V–VII; c, Panels XI–XII; d, Panel III); 100 μm (a, Panels V–VI; d, Panels IV–VI); 0.5 cm (a, Panels VII–VIII; c, Panels XIII–XIV; d, Panels VII–VIII).

Of the 23 WT‐WT zygotes (Figure 1a, top panel), 22 were divided into two‐celled embryos within the first day post‐fusion, with approximately 90% of the two‐celled embryos continuing to divide into embryo‐like structures, cell masses, and calluses, which ultimately regenerated into plantlets (Figure 1a, Panels I–VIII; Table 1), in agreement with a previous report (Uchiumi et al., 2007). In contrast, of the 22 *bbms‐bbms* zygotes (Figure 1b, top panel), 10 degenerated without undergoing first division (Figure 1b, Panels I–II; Table 1) while the remaining 12 showed signs of division. However, the latter zygotes degenerated after a few rounds of cell division (Figure 1b, Panels III–VIII; Table 1). Moreover, the division speed of *bbms‐bbms* zygotes was notably slower compared to that of WT‐WT zygotes (Figure 1a, Panels I–VIII and Figure 1b, Panels III–VIII).

**Table 1:** Developmental profiles of rice zygotes produced by electro‐fusion of gametes isolated from wild type and *bbms* triple mutant rice plants

Gametes used for fusion		No. of produced zygotes	Developmental stage of zygotes/embryos					Developmental rate from zygote to cell mass (%)
Egg cell	Sperm cell		Two‐celled embryo	Globular‐like embryo	Cell mass	Callus	Plantlet	
Wild type	Wild type	23	22	20	20	20	20	87
*bbms*	*bbms*	22	12	10	0	0	0	0
Wild type	*bbms*	23	15	14	8	7	7	35
*bbms*	Wild type	20	16	15	15	11	6	75

Next, we evaluated the impact of *bbms* mutations from the paternal and maternal sides. When 23 of the WT‐*bbms* zygotes from WT egg and *bbms* sperm (Figure 1c, top panel) were examined, 8 exhibited developmental defects at the one‐cell stage without division while 1 and 6 showed developmental defects at the two‐celled embryo and globular‐like embryo structure stages, respectively (Figure 1c, Panels I–II and III–VII; Table 1). Even when these zygotes developed into globular embryo structures, their division speed was noticeably slower compared to WT‐WT zygotes (Figure 1a, Panels I–VIII and Figure 1c, Panels III–VII). Although the remaining eight zygotes formed cell masses, calluses, or plantlets (Figure 1c, Panels VIII–XIV; Table 1), developmental rate of WT‐*bbms* zygotes to cell masses (35%, 8 out of 23 WT‐*bbms* zygotes, Figure 1c; Table 1) was much lower than that of WT‐WT zygotes (87%, Figure 1a; Table 1). In case of reciprocal fusion, *bbms*‐WT zygotes, *bbms* egg fused with WT sperm, (Figure 1d, top panel) resulted in the majority of zygotes exhibiting normal developmental profiles with developmental rate from zygote to cell mass (75%, 15 out of 20) equivalent to WT‐WT zygotes (87%, 20 out of 23, Figure 1a,d; Table 1). Importantly, in contrast to WT‐*bbms* zygotes, the division speed was similar to those of WT‐WT zygotes (Figure 1d, Panels I–VIII; Table 1). When impact of *OsBBMs* mutations were compared between paternal and maternal sides, profound decrease in division speed and developmental rate of zygotes was detected on mutation from paternal side.

### Effects of paternal and maternal *OsBBM* alleles on zygotic division profiles

To precisely compare the early developmental profiles of zygotes with *bbms* mutations in the paternal allele versus the maternal allele, we used gametes isolated from transgenic rice plants expressing H2B‐GFP, termed GFP‐egg and GFP‐sperm, to visualize the nuclei in developing zygotes. This allowed us to count the number of nuclei during zygotic development. Zygotes were produced from three combinations (WT egg‐GFP sperm, GFP egg‐*bbms* sperm, and *bbms* egg‐GFP sperm) and their developmental profiles were monitored at 18, 42, and 66 h after gamete fusion.

**Figure 2:**
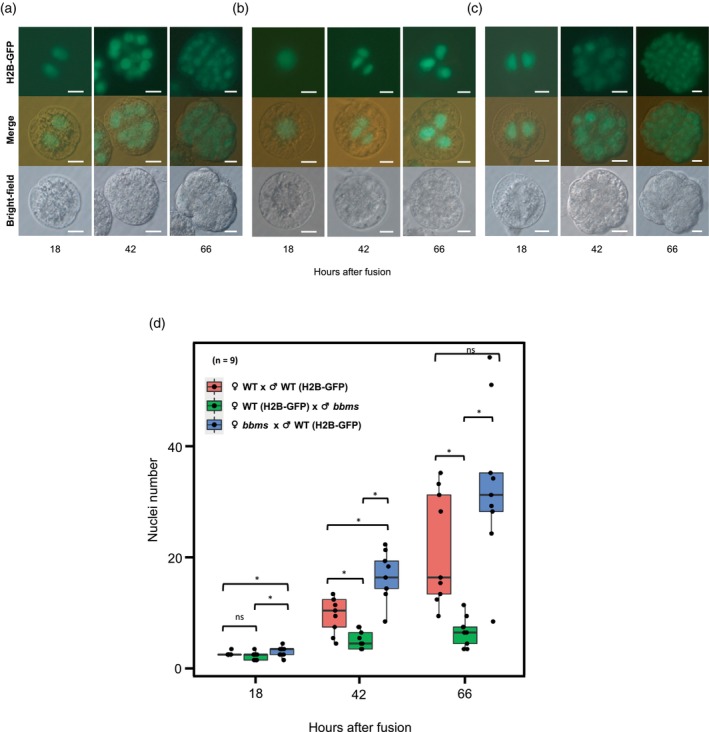
Effects of *OsBBMs* derived from paternal or maternal alleles on the division profiles of rice zygotes. (a–c) Development of zygotes produced by the fusion of an egg cell from wild‐type (WT) rice plants with a sperm cell from WT rice plants expressing H2B‐GFP (a, WT‐GFP zygote), the fusion of an egg cell from WT rice plants expressing H2B‐GFP with a sperm cell from *bbms* triple mutant rice plants (b, GFP‐*bbms* zygote), and the fusion of an egg cell from *bbms* triple mutant rice plants with a sperm cell from WT rice plants expressing H2B‐GFP (c, *bbms*‐GFP zygote). The produced zygotes were cultured, and their developmental and division profiles were observed at 18, 42, and 66 h after gamete fusion. The top, middle, and bottom panels are fluorescence, merged, and bright‐field images, respectively. (d) Average nuclear numbers in early embryos from WT‐GFP zygotes, GFP‐*bbms* zygotes, and *bbms*‐GFP zygotes at 18, 42, and 66 h after gamete fusion. Asterisks indicate significant differences based on analysis of variance (anova), Tukey's honest test (*P* < 0.05); ns = non‐significant (*P* > 0.05); Scale bars: 20 μm.

For WT‐GFP zygotes, all (nine out of nine) zygotes already divided at 18 h after fusion, and the average nuclear numbers were 2.1 at 18 h after fusion. And then, the divided zygotes developed to early embryo‐like structures with average nuclear counts of 9.2 and 21.3 at 42 and 66 h after fusion, respectively (Figure 2a,d). However, when GFP‐*bbms* zygotes (paternal *bbms* mutation) were cultured, three out of nine zygotes remained at the one‐cell stage after 18 h, while the remaining six divided into two‐celled stages (Figure 2b,d). Although nuclear division progressed in the subsequent hours, the average nuclear counts at 42 and 66 h were 4.6 and 6.2, respectively (Figure 2b,d), suggesting that the progression of zygotic division was significantly delayed when male gametes were derived from the *bbms* triple mutant, and that paternal *OsBBMs* play a crucial role in early zygotic division. For *bbms*‐GFP zygotes (maternal *bbms* mutation), the division and proliferation profiles were similar to those of WT‐GFP zygotes (Figure 2c). Surprisingly, the division rate of the *bbms*‐GFP zygotes was higher than that of the WT‐GFP zygotes, with nuclear counts averaging 16.1 and 32.8 nuclei at 42 and 66 h, respectively (Figure 2d).

### Gene expression profiles in zygotes with or without paternal *OsBBMs*

To investigate the molecular mechanisms underlying *OsBBMs*‐dependent zygote development, we conducted transcriptome analyses on zygotes and embryos from the four different gamete combinations at 4, 18, 42, and 66 h post‐fusion. Since defects in paternal *OsBBMs* affect zygotic development and nuclear division at 18 h and 1 day post‐fusion (Figures 1 and 2), transcriptomic analysis was initiated after 4 h, following reports that global *de novo* gene expression in rice zygotes begins around 3–4 h post‐fusion (Ohnishi et al., 2014; Rahman et al., 2019). cDNA synthesis/amplification and library preparation were successfully conducted using five zygotes/embryos with two biological replicates (Figure S2).

**Figure 3:**
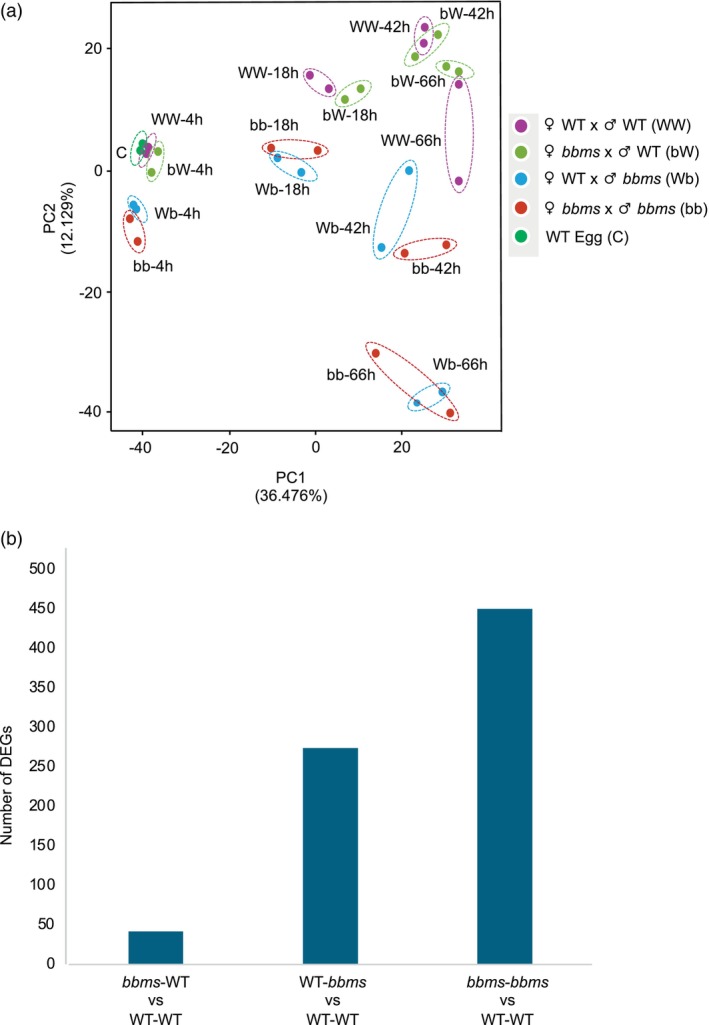
Gene expression profiles in zygotes with or without paternal *OsBBMs*. (a) Principal component analysis of zygotes/embryos at different developmental stages at 4, 18, 42, and 66 h after fusion. (b) The number of differentially expressed genes (DEGs) in *bbms*‐WT, WT‐*bbms*, and *bbms‐bbms* zygotes compared to WT‐WT zygotes at 4 h after fusion.

Principal component analysis (PCA) of the obtained transcriptome data showed that the gene expression profiles were influenced by both the time after gamete fusion and the presence or absence of *bbms* mutations on the paternal side (Figure 3a). These suggest that gene expression profiles progressively change during the early development of rice zygotes, and that *OsBBMs* derived from paternal allele provides more profound effects on machineries in zygotic gene expression than maternal allele‐derived *OsBBMs*.

The number of differentially expressed genes (DEGs) between WT‐WT zygotes and *bbms*‐WT, WT‐*bbms* or *bbms*‐*bbms* zygotes at 4 h after fusion were compared to judge whether gene expression profiles of these zygotes with three combinations are similar or distant to those of WT‐WT zygotes. Although 42 DEGs were detected in *bbms*‐WT zygotes, 274 and 450 DEGs were identified in WT‐*bbms* and *bbms*‐*bbms* zygotes, respectively, that is, 6‐ and 10‐fold higher than those of *bbms*‐WT zygotes, respectively (Figure 3b; Table S1). These suggest that gene expression profile of *bbms*‐WT zygotes, possessing *bbms* mutation in maternal allele, is highly similar to that of WT‐WT zygotes, and that *bbms* mutation in paternal allele affects gene expression profiles in rice zygote. This putative disordered gene expression in WT‐*bbms* zygotes will be related to developmental delay or arrest of WT‐*bbms* zygotes at early zygotic developmental stage (Figures 1c and 2d). Therefore, we next performed WGCNA to detect genes whose expression levels are highly correlated with the presence of *bbms* mutation on paternal allele in rice zygotes.

### Identification of genes whose expression levels were correlated with the presence or absence of paternal *OsBBMs* in zygotes

**Figure 4:**
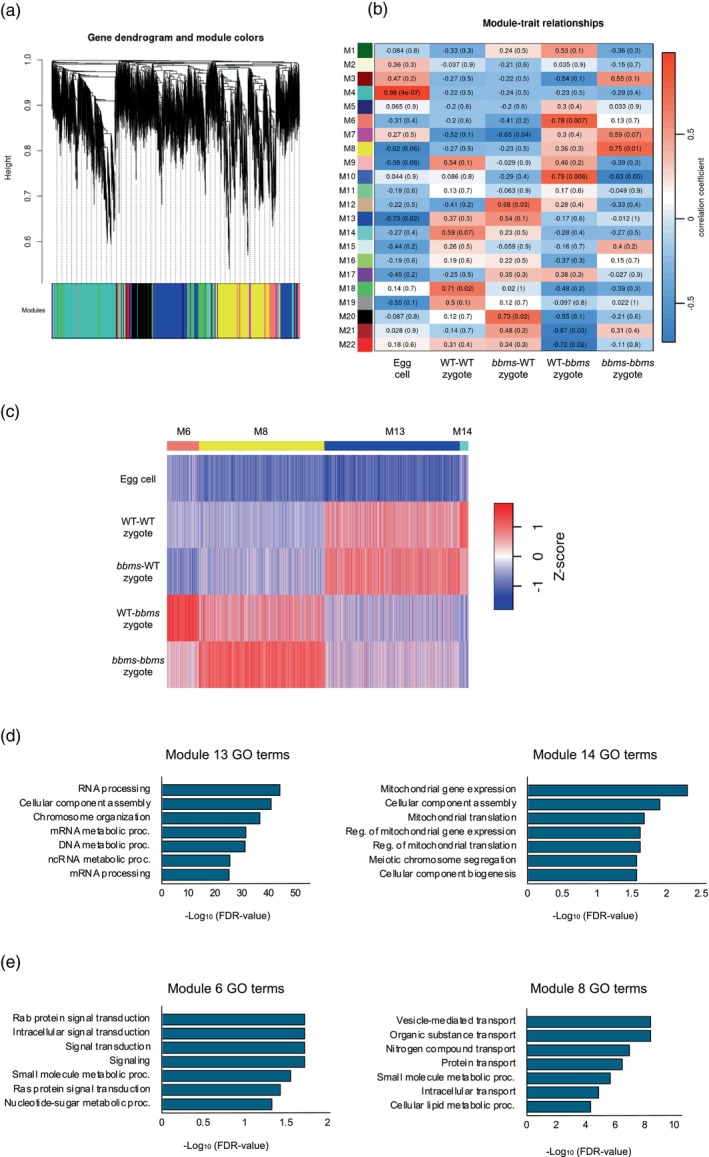
Correlation between the gene modules and gamete combination of zygotes with the presence or absence of paternal *OsBBMs*. (a) Weighted gene co‐expression network analysis (WGCNA) cluster dendrogram showing co‐expression modules. Modules are assigned with distinct colors. (b) Module–trait correlation between the gene modules and gamete combination of zygotes at 4 h after fusion with the presence or absence of paternal *OsBBMs*. The correlation is estimated using the Pearson correlation coefficient method. (c) Heatmap showing expression patterns of four stage‐specific modules across all tested stages. (d, e) Gene Ontology (GO) analysis of the genes that were highly correlated with the presence or absence of paternal *OsBBMs* in rice zygotes.

The WGCNA‐associated hierarchical clustering approach extracted genes with co‐expression patterns among the four types of zygotes at 4 h post‐fusion and egg cells, and categorized the expressed genes into 22 modules (Figure 4a,b). Among these modules, we noticed Modules 6, 8, 13, and 14, as these representative co‐expression modules were highly correlated with the existence of *bbms* mutations on the paternal allele in their zygotic genome. Genes categorized into Modules 13 and 14 were upregulated after gamete fusion (fertilization) in WT‐WT and *bbms*‐WT zygotes, in which paternal *OsBBMs* are functioning; however, the apparent upregulation of these genes was not detectable in WT‐*bbms* and *bbms*‐*bbms* zygotes harboring *bbms* mutations on the paternal allele (Figure 4c; Table S2). These suggest that the expression level of genes in these modules is positively regulated via indirect or direct effects from paternal allele‐derived *OsBBMs* in rice zygotes. Interestingly, genes in Modules 6 and 8 appeared to be mis‐expressed in WT‐*bbms* and/or *bbms*‐*bbms* zygotes, as their low expression levels in WT‐WT and *bbms*‐WT zygotes are approximately equivalent to those in egg cells, and these genes are upregulated only in WT‐*bbms* and *bbms*‐*bbms* zygotes (Figure 4c; Table S2). Genes in these modules may be negatively regulated or suppressed by paternal *OsBBMs* in rice zygotes.

Gene Ontology (GO) enrichment analysis of the genes from Modules 13 and 14 indicated that GO terms related to chromosomal status, such as chromosome organization, DNA metabolic process, RNA processing and translation, were enriched (Figure 4d; Table S3). In Modules 6 and 8, GO terms related to metabolism and biosynthesis, such as small molecule metabolic process, membrane lipid biosynthesis, and nucleotide‐sugar biosynthetic process, were enriched (Figure 4e; Table S3), being consistent with the previous report that genes encoding metabolic pathways are highly downregulated or suppressed in zygote after fertilization (Abiko et al., 2013).

**Figure 5:**
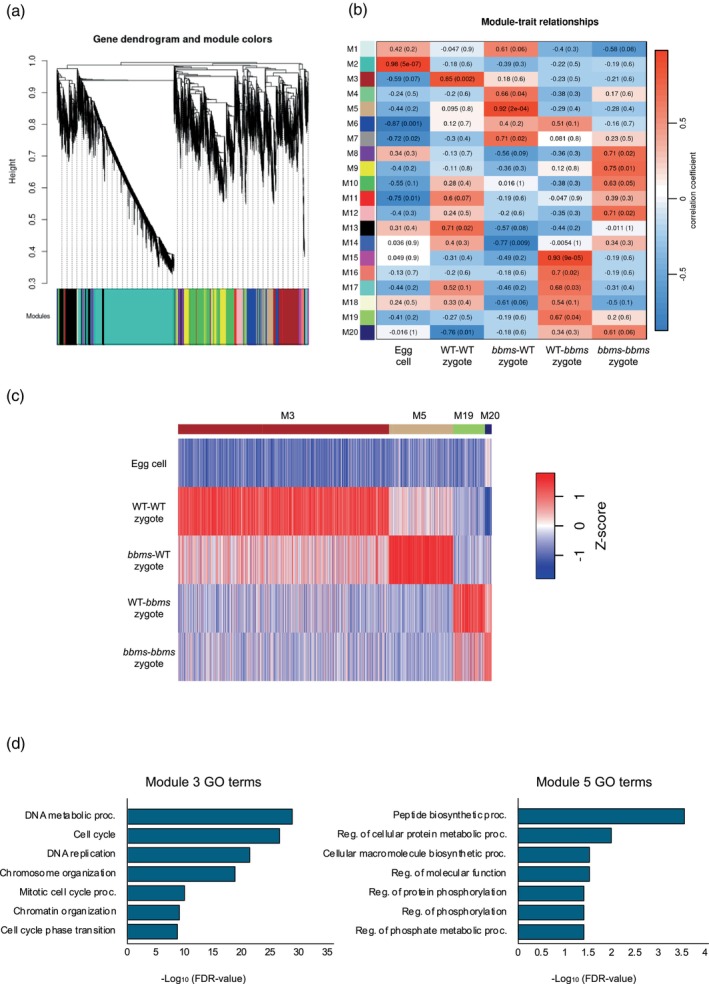
Correlation between the gene modules and gamete combination of zygotes/embryos with the presence or absence of paternal *OsBBMs*. (a) Weighted gene co‐expression network analysis (WGCNA) cluster dendrogram showing co‐expression modules. Modules are assigned with distinct colors. (b) Module–trait correlation between the gene modules and gamete combination of zygotes/embryos at 18 h after fusion with the presence or absence of paternal *OsBBMs*. The correlation is estimated using the Pearson correlation coefficient method. (c) Heatmap showing expression patterns of four stage‐specific modules across all tested stages. (d) Gene Ontology (GO) analysis of the genes that were highly correlated with the presence or absence of paternal *OsBBMs* in rice zygotes.

In addition to zygotes at 4 h after fusion, WGCNA analysis was applied to transcriptome derived from four types of zygotes at 18 h after fusion and egg cells (Figure 5a,b). Twenty modules were created, and among these modules, Modules 3, 5, 19, and 20 were highly correlated with existence of *bbms* mutations on paternal allele on their zygotic genome (Figure 5b). Genes categorized into Modules 3 and 5 were upregulated after gamete fusion (fertilization) in WT‐WT and *bbms*‐WT zygotes, but not in WT‐*bbms* and/or *bbms*‐*bbms* zygotes harboring *bbms* mutations on paternal allele (Figure 5c; Table S4). GO enrichment analysis for genes in these two modules revealed that the GO terms related to cell cycle and chromatin status, such as cell cycle, organelle organization, DNA repair, chromatin organization, and DNA replication, were enriched (Figure 5d; Table S5). In contrast, genes in Modules 19 and 20 appeared to be upregulated only in WT‐*bbms* and *bbms*‐*bbms* zygotes after gamete fusion (Figure 5c). Although GO analysis was conducted for the gene in these modules, no enriched GO term was obtained.

### Allele dependency of genes whose expression levels are possibly regulated by paternal *OsBBMs*

**Figure 6:**
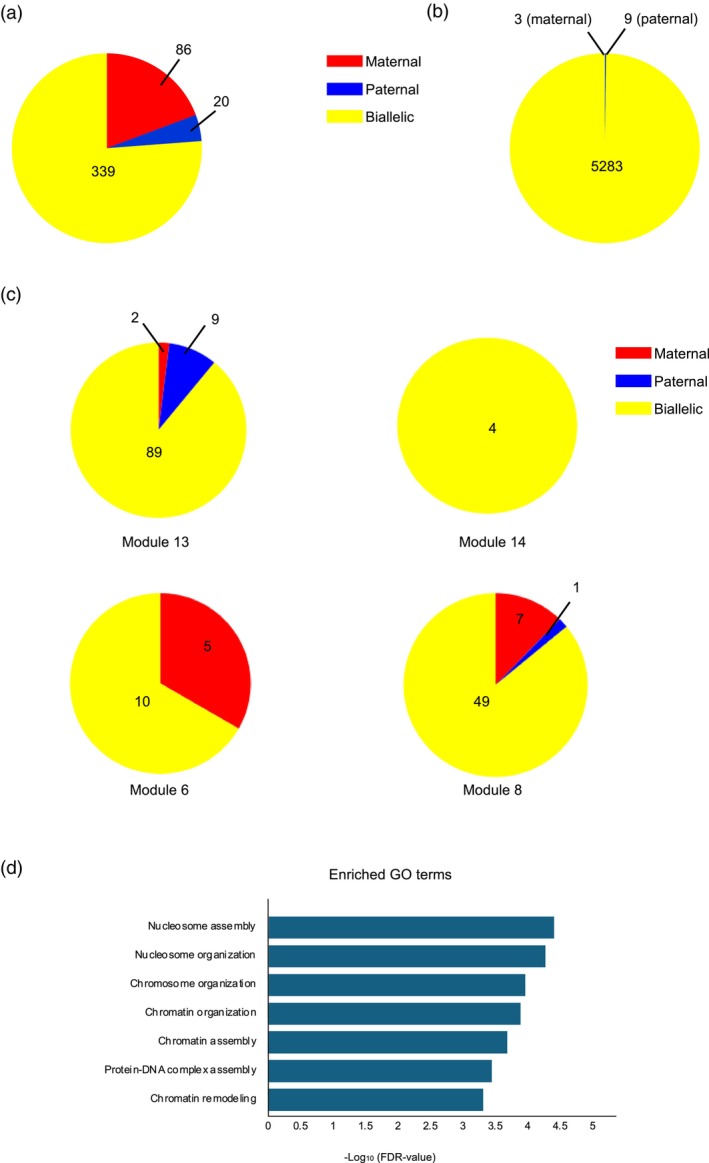
Allele dependency of genes expressed in intersubspecific zygotes (a, b) and genes possibly regulated via paternal *OsBBMs* (c, d). (a) Venn diagram of the 445 genes for which allele dependency was accurately determined in rice zygotes at 4 h after fusion. Twenty, 86, and 339 genes were categorized as paternal monoallelic, maternal monoallelic, and biallelic expressed genes, respectively. (b) Venn diagram of the 5295 genes for which allele dependency was accurately determined in rice zygotes at 18 h after fusion. Nine and three genes were categorized as paternal and maternal preferentially expressed genes, respectively. Five thousand two hundred eighty‐three genes were categorized as biallelic expression. (c) Monoallelic and biallelic genes in (a) were separated into Modules 6, 8, 13, and 14, which were created using the weighted gene co‐expression network analysis (WGCNA) approach in Figure 4. (d) Gene Ontology (GO) analysis of the 89 biallelic genes in Module 13 from panel (c).

After identifying candidate genes whose expression levels were affected by *OsBBMs* from the paternal allele (Figures 4c and 5c; Tables S2 and S4) and considering that *OsBBM1* activates both *OsBBM1* and *OsYUCCA* on the maternal allele (Khanday et al., 2019, 2023), we assessed the allele dependency of these genes. Intersubspecific zygotes between Japonica rice (cv. Nipponbare, NB) and Aus rice (cv. Kasalath, KS) were produced through reciprocal fusion of gametes, and then NB (♀)‐KS (♂) and KS (♀)‐NB (♂) zygotes at 4 and 18 h post‐fusion were subjected to transcriptome analysis and subsequent SNP‐based mapping. On the rice genome, 37 828 genes have been annotated, and SNPs were detected in 30 429 genes between NB and KS (Table S6). This suggests that approximately 80% of the rice genes can be covered in the present SNP‐based mapping. In the 4‐h zygotes, 10 666 genes containing SNPs between NB and KS were expressed (Table S7). To identify genes exhibiting preferential allele dependency, we selected genes whose SNP fragment counts were biased toward the paternal or maternal allele by more than 90% in all four replicates (two replicates of NB‐KS zygotes and two replicates of KS‐NB zygotes) (Tables S8 and S9). Genes showing a fragment ratio on maternal alleles between 0.3 and 0.7 across all four replicates were selected to indicate biallelic expression (Table S10). Among 445 genes where allele dependency was accurately determined, 4.5% (20 genes), 19.3% (86 genes), and 76.2% (339 genes) were categorized as having paternal allele‐dependent, maternal allele‐dependent, and biallelic expression, respectively (Figure 6a; Tables S11–S13). In 18‐h zygotes, among 5295 genes, 99.77% (5283 genes) were categorized as biallelic expression (Figure 6b; Table S14), and only a small fraction of expressed genes, nine genes (0.17%) and three genes (0.06%), were identified as paternal and maternal allele‐preferential genes, respectively (Figure 6b; Tables S15 and S16).

Genes categorized as having paternal or maternal allele‐dependent expression (Figure 6a; Tables S11 and S12) and biallelic expression in 4‐h rice zygotes (Figure 6a; Table S13) were further analyzed to determine their association with Modules 6, 8, 13, and 14 shown in Figure 4b and c (Tables S17–S19), since expression profiles of genes in these modules were possibly affected by the function of *OsBBMs* derived from the paternal allele in 4 h zygotes. Notably, 9 out of 20 genes with paternal allele‐dependent expression were detected in Module 13 (Figure 6c; Table S17), in which genes positively regulated via indirect or direct effects from paternal allele‐derived *OsBBMs* may have accumulated. In contrast, 12 out of 86 genes with maternal allele‐dependent expression were detected in Modules 6 and 8 (Figure 6c; Table S18), in which genes negatively regulated via direct effects of paternal *OsBBMs* may have accumulated. Genes with clear biallelic expression profiles were mainly distributed in Modules 13 and 8 (Figure 6c). Notably, GO terms related to chromosome/chromatin organization, such as nucleosome assembly, chromatin remodeling, and protein–DNA complex assembly, were highly enriched among the 89 biallelic genes in Module 13 (Figure 6d; Table S20). The estimated allele dependency of several genes in Module 13 was confirmed by Sanger sequencing of the PCR‐amplified DNA bands (Figure S3).

## DISCUSSION

*OsBBM1*, belonging to the AP2/ERF transcription factor family, plays an important role in inducing the developmental program of rice zygotes. Mutations in *OsBBM1* have little effect on zygotic development, as it has been suggested that the *BBM*‐related genes *OsBBM2* and *OsBBM3* might act redundantly with *OsBBM1* in rice zygotes (Khanday et al., 2019; Rahman et al., 2019). In this study, using female and male gametes isolated from genome‐edited rice plants harboring knockout mutations in *OsBBM1*, *OsBBM2*, and *OsBBM3*, zygotes of four combinations (WT egg‐WT sperm, *bbms* egg‐*bbms* sperm, *bbms* egg–WT sperm, and WT egg‐*bbms* sperm) were created to address the functions of paternal or maternal *OsBBMs* in zygotic development.

Cytological observations of the developmental and division profiles indicated that mutations in *OsBBMs* on the paternal allele triggered developmental arrest or delay in zygotes, whereas defects in maternal allele‐derived *OsBBMs* had little effect on zygotic development (Figures 1 and 2). Our observations are in line with a previous report that *OsBBM1* derived from paternal alleles plays an essential role in initiating the developmental machinery of rice zygotes (Khanday et al., 2019; Rahman et al., 2019). Surprisingly, *bbms*‐WT zygotes, possessing *OsBBMs* mutations in the maternal allele, appeared to divide and proliferate more actively than zygotes possessing the paternal *OsBBMs* allele (WT‐WT zygotes) (Figure 2). Regarding the developmental profiles of *bbms*‐WT zygotes, we speculated that they would divide with similar or slightly lower efficiency compared to WT‐WT zygotes, since *OsBBM1* is primarily expressed from the paternal allele, and paternal *OsBBM1* activates the *de novo* expression of *OsBBM1* on the maternal allele, resulting in biallelic expression of *OsBBM1* (Khanday et al., 2019; Rahman et al., 2019). The highly active division of *bbms*‐WT zygotes may be explained by our previous findings regarding the effects of the ectopic expression of *OsBBM1* in zygotes (Rahman et al., 2019). When excess *OsBBM1* was expressed in rice WT‐WT zygotes, three out of nine tested zygotes did not divide and gradually degenerated. Importantly, although the remaining six zygotes divided into two‐celled embryos, five of the six two‐celled embryos exhibited developmental arrest. These results suggest that excess *OsBBM1* negatively impacts zygotic development, and that the expression level of *OsBBM1* is strictly maintained at an optimal level. When expression level of *OsBBMs* were monitored in the developing WT‐WT and *bbms*‐WT zygotes that were produced and analyzed in the study, expression level of *OsBBM1* was highest among the *OsBBM1, 2, and 3* both in WT‐WT and *bbms‐*WT zygotes (Table S21). However, expression level of *OsBBM1* in WT‐WT zygotes was much higher than that of *bbms*‐WT zygotes. These results may indicate that *OsBBM1* expression levels in WT‐WT and *bbms*‐WT zygotes are excess and suitable situations for the progression of cellular development, respectively.

In this study, transcriptome analyses were performed on the zygotes to examine how parental *OsBBMs* are involved in the induction of zygotic development. Data analysis revealed that gene expression profiles in rice zygotes were primarily dependent on the presence or absence of *bbms* mutations on the paternal side (Figure 3a), indicating that the paternal allele of *OsBBMs* had a more significant impact on zygotic gene expression than the maternal allele (Figure 3b). Through WGCNA and subsequent monitoring of gene expression categorized into several modules, we detected genes whose expression levels were commonly increased in both WT‐WT and *bbms*‐WT zygotes after gamete fusion, but not in WT‐*bbms* and *bbms‐bbms* zygotes (Figures 4 and 5). Among these genes, GO terms associated with chromosome organization and cell division, including cell cycle, chromosome organization, DNA replication, chromatin organization, DNA metabolic processes, and RNA processing, were enriched (Figures 4d and 5d; Tables S3 and S5). These are consistent with the previous reports that reorganization of chromatin and *de novo* gene expression in the zygotic nucleus and subsequent active protein synthesis appeared to occur during the early stage of rice zygotes (Ohnishi et al., 2014). In addition, Zhao et al. (2019) reported that GO terms of cell cycle, chromosome organization, and cell division were enriched in upregulated genes in Arabidopsis zygotes after fertilization, suggesting that similar or conserved cellular machineries function in developing zygotes both in Arabidopsis and rice. Therefore, to estimate genes functioning in the process of cell cycle, chromosome organization, or cell division in rice zygotes, genes were extracted from modules 6, 8, 13, and 14 in 4 h zygotes and modules 3 and 5 in 18 h zygotes, as the mis‐expression of these genes will have a negative impact on zygotic development, resulting in the developmental delay or arrest of zygotes lacking paternal *OsBBMs*.

Regarding chromosome/chromatin organization, we noted a gene in Module 13 (Figure 4), *Os03g0165266*, which encodes the chromatin remodeling factor *OsCHR730*. This gene is a member of the Snf2 family proteins, which can modulate developmental phase through DNA replication, transcription, and/or DNA repair (Guo et al., 2022). Moreover, 42 histone‐related genes were detected (Table S2) in Module 13, providing a possibility that OsCHR730 and these histone‐related proteins are involved in chromatin remodeling during zygotic development (Kawashima & Berger, 2014; Zhou et al., 2019). Regarding cell cycle properties, the increased expression levels of *Os12g0127400* (Table S3), which encodes a cyclin‐like F‐box domain‐containing protein, might enable early zygotes to properly progress through the cell cycle and somatic embryogenesis (Boycheva et al., 2015). Interestingly, among genes related to cell division, we detected 11 genes encoding cyclin‐like F‐box domain‐containing proteins in Modules 3 and 5 (Figure 5; Table S4). As the cyclin‐like F‐box was shown to control the level of the G2/M transition‐specific gene cyclin B1:1 (*CYCB1:1*) (Boycheva et al., 2015), it is suggested that under the influence of paternal *OsBBMs*, the multiplication of cyclin‐like F‐box protein gene expression activates zygotes to proceed with the M‐phase and divide into two‐celled embryos. In addition to the cyclin‐like F‐box, the cyclin A/B/D/E domain‐containing protein gene (*Os07g0620800*) was highly expressed in zygotes harboring paternal *OsBBMs* (Module 3 in Figure 5c). A well‐characterized microtubule‐severing ATPase KATANIN, encoded by *Os01g0683100* in rice, which is known to be involved not only in mitosis and cell division orientation but also in plant development and differentiation (Luptovčiak et al., 2017), was also upregulated (Table S4). These findings suggest that the gradual accumulation of cell/tissue differentiation‐related proteins, such as the cyclin A/B/D/E domain‐containing protein and KATANIN (Jiang et al., 2024; Luptovčiak et al., 2017; Sato et al., 2024), might be initiated primarily in zygotes 18 h after fusion, in which paternal *OsBBMs* are active. In addition, a gene encoding a SUMO domain‐containing protein (*Os01g0607300*) may also contribute to zygotic division, as proper cell division is partly achieved through the activation of the SUMOylation system‐mediated developmental processes (Ghimire et al., 2020; Srivastava et al., 2021).

Auxin, an important plant growth regulator, is widely known to play crucial roles in various developmental processes in plants, particularly in early embryogenesis (Weijers et al., 2005; Yuan et al., 2017), and has recently been reported to be directly activated by *OsBBMs* in calli derived from the rice scutellum (Khanday et al., 2023). In our transcriptome data, genes involved in the auxin‐mediated cellular machinery were significantly upregulated in zygotes expressing functional *OsBBMs* from paternal alleles (Module 3 in Figure 5c). For instance, a gene encoding auxin response factor (ARF) 24 (*Os12g0479400*) was found to be upregulated in developing zygotes (Table S4). ARF has been demonstrated to function in auxin‐dependent growth, with embryo organization being partially coordinated by cell expansion and elongation via a cell wall remodeling mechanism in Arabidopsis (Waller et al., 2002; Weijers et al., 2005). The regulatory function of paternal *OsBBMs* in cell growth through the re‐establishment of the cell wall is also supported by the upregulation of *Os03g0416300*, which encodes a COBRA‐like protein, a key regulator in cell expansion and cell wall biosynthesis (Dai et al., 2009). Taken together, these results suggest that *OsBBMs* derived from paternal alleles induce cell wall reconstruction, leading to cell growth and division in early embryos in an auxin‐dependent manner.

Next, we examined the allele dependency of genes expressed in rice zygotes to investigate the synergistic relationship between the paternal and maternal genomes (alleles) using SNP‐based transcriptome analyses of inter‐subspecific rice zygotes. In these zygotes, we identified 20 and 86 genes with monoallelic expression that were distinct from the paternal and maternal alleles, respectively (Figure 6; Tables S11 and S12). Importantly, 9 out of the 20 genes with preferential expression from the paternal allele were detected in Module 13, where genes that were upregulated, possibly via paternal *OsBBMs*, were categorized (Figure 4b,c). Additionally, 12 out of the 86 genes preferentially expressed from the maternal allele were detected in Modules 6 and 8, where genes that were misexpressed in rice zygotes due to mutations in paternal *OsBBMs* were categorized (Figure 4b,c). These results suggest that *OsBBMs* derived from paternal alleles in zygotes are required to induce and suppress the expression of several genes from paternal and maternal alleles, respectively; however, whether *OsBBMs* regulate the expression of these monoallelic genes in a direct or indirect manner remains unclear. Among the nine possible paternal genes in Module 13, a notable gene, *Os10g0519800*, encoding the F‐box‐type E3 ubiquitin ligase (*FB*) *DUF48*, is worth mentioning, as it has been reported that *FB‐DUF48* is expressed from the paternal allele in the rice endosperm and positively regulates the size of embryos as well as endosperms (Yuan et al., 2017). Paternally expressed *FB‐DUF48* in early zygotes may also be involved in the proper developmental progression of zygotes.

In addition to genes exhibiting monoallelic expression, genes related to chromosome/chromatin organization and assembly were highly enriched among the 89 biallelic genes in Module 13 (Figure 6c,d). In the list of these 89 genes (Table S19), we detected six histone genes (*Os01g0153300, Os09g0553100, Os03g0279000, Os01g0835900, Os04g0583600, Os10g0418000*) and one histone chaperone‐related gene (*Os04g0321600*), which facilitate the chromatin transcription (FACT) complex subunit SPT16. SPT16 is thought to be involved in DNA replication, repair, recombination, and transcription through histone modification and incorporation into the nucleosome (Corpet & Almouzni, 2009; Mehrotra et al., 2011). The upregulation of genes for histone components and the FACT complex via paternal *OsBBMs* in zygotes suggests that the histone chaperone complex SPT16 plays a role in reprogramming chromatin architecture and regulating gene expression profiles through histone modification, thereby facilitating the progression of early development of rice zygotes.

**Figure 7:**
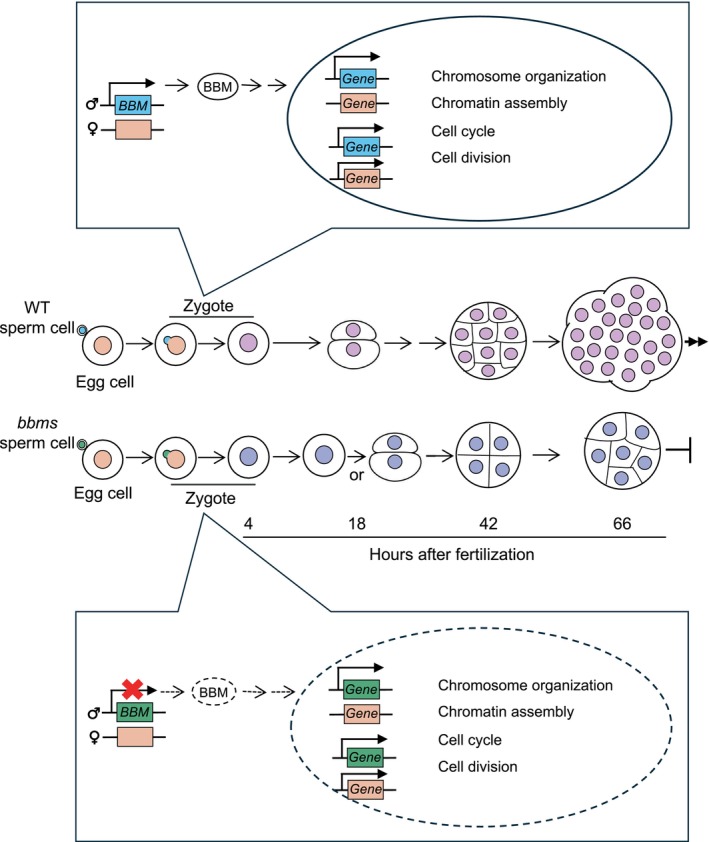
Schematic illustration of developmental progression in rice zygotes via *OsBBMs* derived from the paternal allele. In rice zygotes arising from the union of gametes, the zygotic nucleus is formed through karyogamy, during or after which *de novo* gene expression is initiated from the zygotic genome, leading to precise and active zygotic development (Ohnishi et al., 2014). Subsequently, the zygote divides into a two‐celled embryo through the reorganization of cellular polarity, and the two‐celled embryo further develops into a globular‐like embryo structure and cell mass (Uchiumi et al., 2007; Sato et al., 2010). The presence of the paternal allele of *OsBBMs* allows for the proper early development of zygotes and embryos due to the appropriate expression of genes related to cellular machinery, such as chromosome organization, chromatin assembly, cell cycle, and cell division. However, in zygotes harboring *bbms* mutations in the paternal allele, these genes are negatively regulated, suppressed, or misexpressed, resulting in developmental delays or defects.

In this study, we detected a significant delay in the development of rice zygotes possessing *bbms* mutations in the paternal allele, but not in zygotes harboring *bbms* mutations in the maternal allele. This developmental delay or defect could be attributed to unsuccessful progression of early developmental cellular machinery in zygotes, including *de novo* gene expression, cell cycle restart, and cellular polarity reorganization. These findings suggest that the expression levels of many genes possibly related to these zygotic cellular events are upregulated via *OsBBMs* derived from paternal alleles, likely through direct or indirect interactions (Figure 7). To elucidate the mechanisms underlying the sequential and/or synergistic functions between paternal *OsBBMs* and the paternal *OsBBMs*‐dependent genes, further investigations of putative target genes are underway in our laboratories.

## MATERIALS AND METHODS

### Plant materials

*Oryza sativa* L. cv. Nipponbare and cv. Kasalath were grown in an environmental chamber (K30‐7248; Koito Industries, Yokohama, Japan) at 26°C with a 13‐h light/11‐h dark photoperiod. Transformed rice plants (cv. Nipponbare) expressing the histone H2B‐GFP fusion protein were prepared as previously described (Abiko et al., 2013).

### Production of genome‐edited rice plants for *OsBBM1* , *OsBBM2* , and *OsBBM3*

The CRISPR/ Cas9 vector for OsBBM1, OsBBM2, and OsBBM3 was constructed using method described by Mikami et al. (2015). The target sequences of three BBM genes were selected as previously described in Khanday et al. (2019); BBM1: 5′‐GGAGGACTTCCTCGGCATGC‐3′, BBM2: 5′‐GTATGCAATATACTCCTGCC‐3′, and BBM3: 5′‐GACGGCGGGAGCTGATCCTG‐3′. The resulting binary vector plasmid was introduced into Agrobacterium tumefaciens EHA105, and transgenic rice plants were prepared according to Hiei et al., 1994. Transgenic plants of BBM1 (+/−) BBM2 (−/−) BBM3 (−/−) were obtained, and their progeny possessing homozygous knockout mutations in all three loci, BBM1 (−/−) BBM2 (−/−) BBM3 (−/−), were used as *bbms* triple mutant (*bbm1bbm2bbm3*) in the present study.

### Isolation and electrofusion of gametes

Egg and sperm cells were isolated from rice flowers as previously described (Uchiumi et al., 2006), and electrofusion with the isolated gametes was conducted for zygote production as previously reported (Uchiumi et al., 2007).

### Microscopy

The gametes, zygotes, and embryo‐like structures were observed using an IX‐71 inverted fluorescence microscope (Olympus, Tokyo, Japan). The fluorescence signal for H2B‐GFP was detected at excitation and emission wavelengths of 460–490 and 510–550 nm, respectively (U‐MWIBA2 mirror unit; Olympus). Digital images of gametes, zygotes, and the resulting multicellular structures and cell masses were captured using a cooled charge‐coupled device camera (Penguin 600CL; Pixcera, San Jose, CA, USA) with InStudio software (Pixcera).

### Preparation of lysates from gametes, zygotes, and embryos for RNA‐seq analysis

Using gametes isolated from Nipponbare (WT) and *bbms* triple mutant rice plants, zygotes with four gamete combinations (WT egg‐ WT sperm, *bbms* egg‐*bbms* sperm, WT egg‐*bbms* sperm, and *bbms* egg‐WT sperm) were prepared by electrofusion, as described above. The resulting zygotes were cultured for 4, 18, 42, and 66 h and subjected to cDNA synthesis and amplification with two independent biological replicates, as described below. After culture, zygotes and early embryos were transferred to droplets of mannitol solution adjusted to 450 mOsmol kg^−1^ H_2_O on coverslips, and the washing procedure was performed three or four times by transferring the cells to fresh droplets of mannitol solution. After washing, the zygotes were transferred to a lysis buffer supplied by the SMART‐Seq HT Kit (Takara Bio, Shiga, Japan). The resulting lysates were used for cDNA synthesis directly or stored at −80°C until use.

### cDNA synthesis, library preparation, and RNA‐Seq

cDNA and library preparation were performed as previously described (Deushi et al., 2021). Briefly, cDNA was synthesized and amplified from cell lysates using a SMART‐Seq HT Kit (Takara Bio) according to the manufacturer's instructions. The amplified cDNAs were purified using Agencourt AMPure XP (Beckman Coulter, Brea, CA, USA). The quality and quantity of the purified cDNA were assessed using a Qubit 3 Fluorometer with a Qubit dsDNA HS Assay Kit (Thermo Fisher Scientific, Waltham, MA, USA) and an Agilent 2100 BioAnalyzer with a high‐sensitivity DNA chip (Agilent Technologies, Santa, Clara, CA, USA). Sequencing libraries were prepared from the amplified cDNA using the Nextera XT DNA Library Prep Kit (Illumina, San Diego, CA, USA) according to the SMART‐Seq HT Kit instructions, after which they were purified using Agencourt AMPure XP. After checking the quality and quantity of the purified libraries using the aforementioned procedures for purified cDNA, the prepared libraries were sequenced on an Illumina NovaSeq X platform (Illumina) at Macrogen‐Japan (Tokyo, Japan) to produce 150‐bp paired‐end reads.

### Transcriptome data analysis

The quality of Illumina reads was evaluated using FastQC (v0.12.1; Simon, 2010). Preprocessing of the reads was conducted using fastp (v0.23.4; Chen et al., 2018) to remove adapters, poly‐A tails, and low‐quality sequences. The preprocessed reads were mapped to the reference NB genome sequence (Os‐Nipponbare‐Reference‐IRGSP‐1.0), available in the Rice Annotation Project Database (RAP‐DB) (Kawahara et al., 2013; Sakai et al., 2013), using HISAT2 (v2.2.1; Kim et al., 2015; Pertea et al., 2016). Reads counts and transcripts per million (TPM) were calculated using StringTie (v2.2.1; Pertea et al., 2015, 2016). The DEGs between the zygotes at different time points were identified by comparing read counts using Tag Count Comparison (TCC) (Sun et al., 2013), which is part of the R software. GO enrichment analysis was performed using the hypergeometric test in ShinyGO (v0.8; Ge et al., 2020).

### Weighted gene co‐expression network analysis

A WGCNA was performed to identify co‐expression networks by comparing TPM using a R package, WGCNA (Langfelder & Horvath, 2008, 2012). The median absolute deviation (MAD) of each gene was calculated as a robust measure of variability. Genes were then sorted based on MAD values, and the top 10 000 ranked genes were used for WGCNA. A weighted network adjacency matrix was created for each time point with soft thresholding powers of 22 and 16 for 4 and 18 h after fusion, respectively, which were determined according to the scale‐free topology criterion. Next, an average linkage hierarchical clustering tree was constructed using topological overlap‐based dissimilarity for each pair of genes. A dynamic hybrid tree‐cut algorithm was used to cut the hierarchical clustering tree and define the modules. A minimum module size of 30 and a height cut of 0.4 for 4 h, and 0.24 for the other time points, were used to merge the modules. To identify the modules that were significantly associated with each developmental stage, the Pearson correlation (*R*) and Student's asymptotic *P*‐value (*P*) between each module and developmental stage were calculated.

### RNA‐sequencing of intersubspecific zygotes and subsequent transcriptome data analyses

Gametes were isolated from flowers of Nipponbare (NB) and Kasarath (KS) rice plants as described above. Intersubspecific zygotes were prepared by electrofusion of a NB egg cell and a KS sperm cell (NB × KS; NK zygote) or a KS egg cell and a NB sperm cell (KS × NB; KN zygote). After 4 or 18 h, the zygotes and early embryos were subjected to RNA sequencing as above. To estimate the parental origin of the transcripts, high‐throughput sequencing reads from the whole KS genome (DRR008446 and DRR008447) (Sakai et al., 2014) were used to detect SNPs between cv. KS and cv. NB. Raw sequencing reads were filtered to remove adapters and low‐quality bases, and reads shorter than 20 bp were excluded using fastp version 0.23.4 (Chen et al., 2018). The filtered reads were mapped to reference NB genome sequences (Kawahara et al., 2013; Os‐Nipponbare‐Reference‐IRGSP‐1.0) using the Burrows–Wheeler Aligner (BWA) version 0.7.17 (Li & Durbin, 2009). SNPs were called and annotated using the Genome Analysis Toolkit (GATK) version 4.3.0.0 (McKenna et al., 2010) and SnpEff (Cingolani et al., 2012), respectively. Only homozygous genotypes of the KS allele on the biallelic SNPs were defined as KS‐specific SNPs, and only these SNPs were used for subsequent allele‐specific expression analyses.

### Allele‐specific expression analyses

Raw sequencing reads from NB × KS and KS × NB zygotes with two biological replicates at each time point were first quality‐filtered using fastp version 0.23.4 (Chen et al., 2018) and then mapped to reference NB genome sequences (IRGSP‐1.0) using STAR (version 2.7.10) (Dobin et al., 2013). The reads at each SNP site were counted using the GATK ASEReadCounter (Castel et al., 2015). Allelic imbalance (AI) was calculated per gene as the total number of reads on the maternal alleles (i.e., NB alleles for NB × KS zygotes and KS alleles for KS × NB zygotes) divided by the total number of reads on both the NB (reference) and KS (alternate) alleles. We selected genes whose read fragments to SNPs represented more than five counts in all four replicates and grouped these genes into the following three categories based on AI threshold: (1) maternal‐biased expressed genes with AI > 0.9 in all replicates, (2) paternal‐biased expressed genes with AI < 0.1 in all replicates, and (3) biparental expressed genes with 0.3 ≤ AI ≤ 0.7 in all replicates.

### PCR using cDNA templates for zygotes and SNP verification

cDNA of intersubspecific zygotes between NB and KS was synthesized as described above. For PCR, 1 μl of cDNA (200 pg μl^−1^) was used as the template in a 20 μl PCR reaction with 0.3 μM of primers using KOD‐FX DNA polymerase (Toyobo, Osaka, Japan) as follows: 35 cycles of 98°C for 10 sec, 55°C for 30 sec, and 68°C for 30 sec. The expression of the ubiquitin gene (Os02g0161900) was monitored as an internal control. Primer sequences used for PCR analyses were listed in Table S22. For SNP determination, amplified DNA bands by the above were purified using the PCR purification kit (Qiagen, Valencia, CA, USA), and then sequenced.

## AUTHOR CONTRIBUTIONS

NA, YS, and TO came up with the study idea; NA, KR, and TO designed the experiments; TT created the triple mutant rice plants (*bbms*); NA performed most of the experiments; AS performed bioinformatic analysis; AK performed statistical analyses; TO supervised the project; NA and TO conceived the project and wrote the article.

## CONFLICT OF INTEREST

The authors have not declared a conflict of interest.

## Supporting information


**Figure S1.** CRISPR/Cas9‐mediated triple mutant rice plants. Triple mutations in *BBM1*, *BBM2*, and *BBM3* with the nucleotide homozygous insertion shown in red.
**Figure S2.** Electropherograms of cDNAs and libraries from rice zygotes and embryos. Synthesized and amplified cDNAs (a) and prepared libraries (b) were analyzed using the Agilent 2100 Bioanalyzer with a High Sensitivity DNA chip. FU, fluorescence absorption units. WT‐WT zygote, WT egg–WT sperm; *bbms*‐*bbms* zygote, *bbms* egg‐*bbms* sperm; WT‐*bbms* zygote, WT egg‐*bbms* sperm; *bbms*‐WT zygote, *bbms* egg‐WT sperm.
**Figure S3.** Confirmation of expression profiles and determination of allele dependency of genes expressed in intersubspecific zygotes. Intersubspecific zygotes were prepared by reciprocal electro‐fusion of gametes from NB and KS plants, and cDNAs from these intersubspecific zygotes at 4 h after gamete fusion were used for PCR to verify expression in zygotes (a–c) and allele dependency of genes with paternal (d), maternal (e), or biallelic (f) expression via Sanger sequencing of the PCR‐amplified DNA bands in panel (a, b, and c), respectively. Closed circles on the nucleotide sequence indicate the polymorphism between NB and KS, and the polymorphic position on the chromatogram is also indicated by closed circles. PCR‐amplified DNA band images for control PCR reaction with ubiquitin primer in panels a and b show same pattern, as the procedures of genome PCR using primer sets for panels (a and b) were conducted at the same time together with primer set to ubiquitin gene.

**Table S1.** Number of DEGs in *bbms*‐WT, WT‐*bbms*, and *bbms*‐*bbms* zygotes compared to WT‐WT zygotes at 4 HAF.
**Table S2.** Identified genes from Modules 13, 14, 6, and 8 whose expression levels were putatively upregulated in WT‐WT and *bbms*‐WT zygotes at 4 HAF.
**Table S3.** GO terms enriched from the genes in Modules 13, 14, 6, and 8 that were upregulated in WT‐WT and *bbms*‐WT zygotes at 4 HAF.
**Table S4.** Identified genes from Modules 3 and 5 whose expression levels were putatively upregulated in WT‐WT and *bbms*‐WT zygotes at 18 HAF.
**Table S5.** GO terms enriched from the genes in Modules 3 and 5 that were upregulated in WT‐WT and *bbms*‐WT zygotes at 18 HAF.
**Table S6.** Number of SNPs in rice genes between Nipponbare (NB) and Kasalath (KS) rice.
**Table S7.** Allele dependency and expression profiles of genes in rice zygotes at 4 HAF.
**Table S8.** Genes in rice zygotes with paternal allele‐dependent expression at 4 HAF.
**Table S9.** Genes in rice zygotes with maternal allele‐dependent expression at 4 HAF.
**Table S10.** Genes in rice zygotes with biallelic expression at 4 HAF.
**Table S11.** Genes with preferential expression from the paternal allele at 4 HAF.
**Table S12.** Genes with preferential expression from the maternal allele at 4 HAF.
**Table S13.** Genes with biallelic expression at 4 HAF.
**Table S14.** Genes with biallelic expression at 18 HAF.
**Table S15.** Genes with preferential expression from the paternal allele at 18 HAF.
**Table S16.** Genes with preferential expression from the maternal allele at 18 HAF.
**Table S17.** Genes with preferential expression from the paternal allele in Modules 13 and 8 at 4 HAF.
**Table S18.** Genes with preferential expression from the maternal allele in Modules 13, 6, and 8 at 4 HAF.
**Table S19.** Genes with biallelic expression in Modules 13, 14, 6, and 8 at 4 HAF.
**Table S20.** GO terms enriched from biallelic expressed genes in Modules 13 at 4 HAF.
**Table S21.** Expression levels of *OsBBM1, 2*, and *3* in rice zygotes produced by electro‐fusion of gametes isolated from wild type and *bbms* triple mutant rice plants.
**Table S22.** Primers used for PCR.

## Data Availability

The data that supports the findings of this study are available in the supplementary material of this article. In addition sequencing data were deposited in the NCBI database as ID PRJNA1253340.
