# Supplementary material for: Role of paternal Oryza sativa Baby Booms (OsBBMs) in initiating de novo gene expression and regulating early zygotic development in rice
Source: Plant J. 2025 Jun 26;122(6):e70305. doi: 10.1111/tpj.70305 (PMC12201997; doi:10.1111/tpj.70305)
Supplement: Supplementary file 1 — Figure S1. CRISPR/Cas9‐mediated triple mutant rice plants. Triple mutations in BBM1, BBM2, and BBM3 with the nucleotide homozygous insertion shown in red. Figure S2. Electropherograms of cDNAs and libraries from rice zygotes and embryos. Synthesized and amplified cDNAs (a) and prepared libraries (b) were analyzed using the Agilent 2100 Bioanalyzer with a High Sensitivity DNA chip. FU, fluorescence absorption units. WT‐WT zygote, WT egg–WT sperm; bbms‐bbms zygote, bbms egg‐bbms sperm; WT‐bbms zygote, WT egg‐bbms sperm; bbms‐WT zygote, bbms egg‐WT sperm. Figure S3. Confirmation of expression profiles and determination of allele dependency of genes expressed in intersubspecific zygotes. Intersubspecific zygotes were prepared by reciprocal electro‐fusion of gametes from NB and KS plants, and cDNAs from these intersubspecific zygotes at 4 h after gamete fusion were used for PCR to verify expression in zygotes (a–c) and allele dependency of genes with paternal (d), maternal (e), or biallelic (f) expression via Sanger sequencing of the PCR‐amplified DNA bands in panel (a, b, and c), respectively. Closed circles on the nucleotide sequence indicate the polymorphism between NB and KS, and the polymorphic position on the chromatogram is also indicated by closed circles. PCR‐amplified DNA band images for control PCR reaction with ubiquitin primer in panels a and b show same pattern, as the procedures of genome PCR using primer sets for panels (a and b) were conducted at the same time together with primer set to ubiquitin gene. [file TPJ-122-0-s002.pdf]

Supplementary Figures

CRISPR/Cas9-mediated mutations in *BBM1*, *BBM2* and *BBM3* in rice

***bbm1*: Homozygous with 1 bp insertion**

```
BBM1_WT    TTCCTCGGCAT-GCAGGTGCAG
bbm1_M     TTCCTCGGCATTGCAGGTGCAG
```

***bbm2*: Homozygous with 1 bp insertion**

```
BBM2_WT    ATGACCAGGC-AGGAGTATATT
bbm2_M     ATGACCAGGCTAGGAGTATATT
```

```
BBM3_WT    TCTCCCCGCAG-GATCAGCTCC
bbm3_M     TCTCCCCGCAGAGATCAGCTCC
```

Figure S1. CRISPR/Cas9-mediated triple mutant rice plants.

Triple mutations in *BBM1*, *BBM2* and *BBM3* with the nucleotide homozygous insertion shown in red.

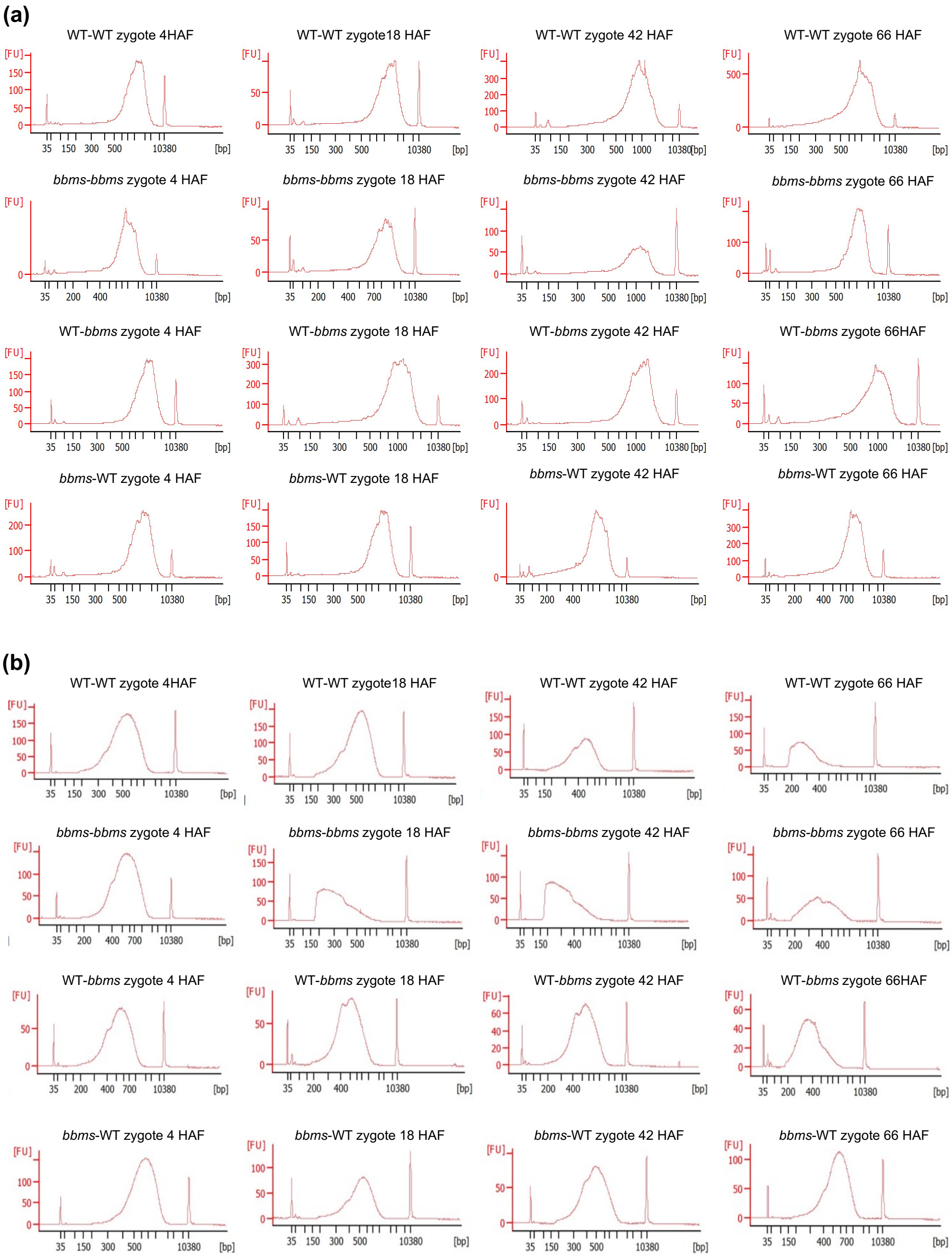

Figure S2. Electropherograms of cDNAs and libraries from rice zygotes and embryos.

Synthesized and amplified cDNAs **(a)** and prepared libraries **(b)** were analyzed using the Agilent 2100 Bioanalyzer with a High Sensitivity DNA chip. FU, fluorescence absorption units. WT-WT zygote, WT egg –WT sperm; *bbms-bbms* zygote, *bbms* egg-*bbms* sperm; WT-*bbms* zygote, WT egg-*bbms* sperm; *bbms*-WT zygote, *bbms* egg-WT sperm.

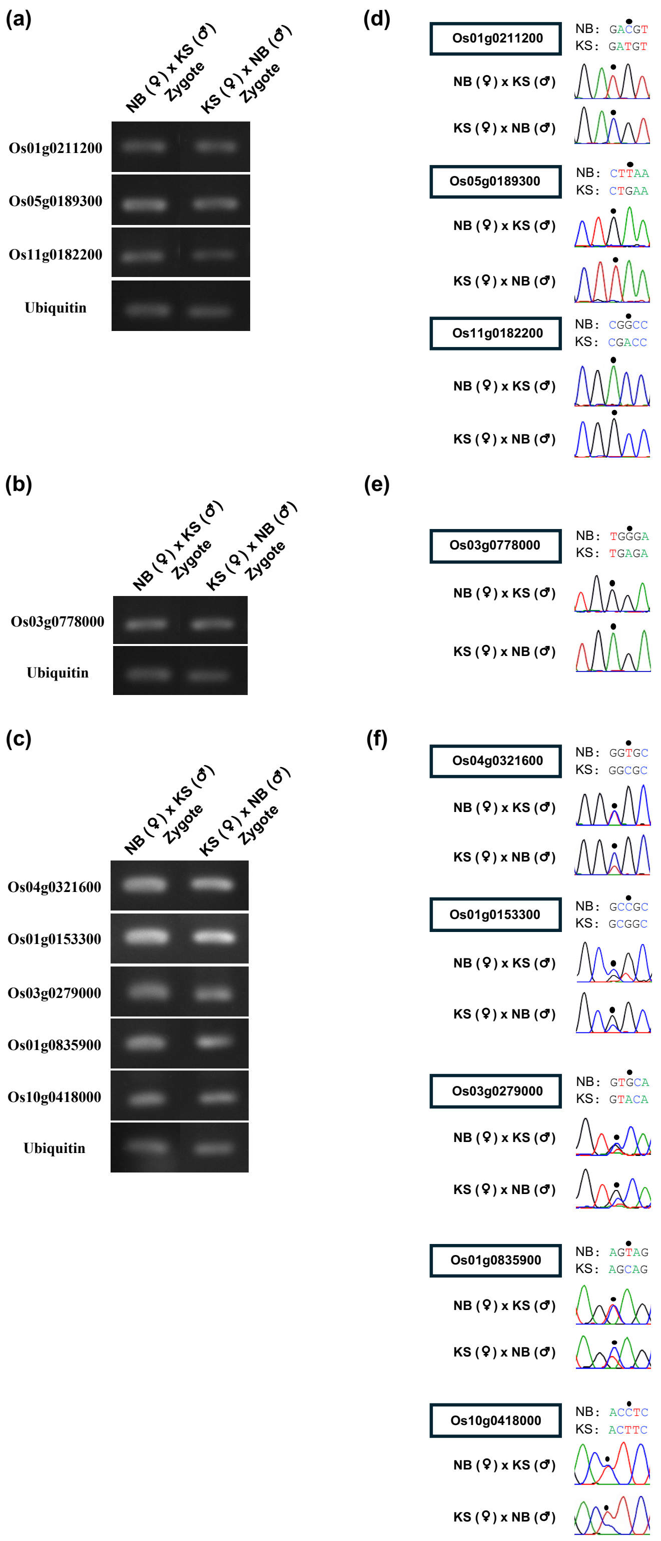

Figure S3. Confirmation of expression profiles and determination of allele dependency of genes expressed in intersubspecific zygotes.

Intersubspecific zygotes were prepared by reciprocal electro-fusion of gametes from NB and KS plants, and cDNAs from these intersubspecific zygotes at 4 h after gamete fusion were used

for PCR to verify expression in zygotes **(a-c)** and allele dependency of genes with paternal **(d),** maternal **(e)** or biallelic **(f)** expression via Sanger sequencing of the PCR-amplified DNA bands in panel **a, b** and **c,** respectively. Closed circles on the nucleotide sequence indicate the polymorphism between NB and KS, and the polymorphic position on the chromatogram is also indicated by closed circles. PCR-amplified DNA band images for control PCR reaction with ubiquitin primer in panels a and b show same pattern, as the procedures of genome PCR using primer sets for panels a and b were conducted at the same time together with primer set to ubiquitin gene.
