# Supplementary material for: Role of paternal Oryza sativa Baby Booms (OsBBMs) in initiating de novo gene expression and regulating early zygotic development in rice
Source: Plant J. 2025 Jun 26;122(6):e70305. doi: 10.1111/tpj.70305 (PMC12201997; doi:10.1111/tpj.70305)
Supplement: Supplementary file 2 — Table S1. Number of DEGs in bbms‐WT, WT‐bbms, and bbms‐bbms zygotes compared to WT‐WT zygotes at 4 HAF. Table S2. Identified genes from Modules 13, 14, 6, and 8 whose expression levels were putatively upregulated in WT‐WT and bbms‐WT zygotes at 4 HAF. Table S3. GO terms enriched from the genes in Modules 13, 14, 6, and 8 that were upregulated in WT‐WT and bbms‐WT zygotes at 4 HAF. Table S4. Identified genes from Modules 3 and 5 whose expression levels were putatively upregulated in WT‐WT and bbms‐WT zygotes at 18 HAF. Table S5. GO terms enriched from the genes in Modules 3 and 5 that were upregulated in WT‐WT and bbms‐WT zygotes at 18 HAF. Table S6. Number of SNPs in rice genes between Nipponbare (NB) and Kasalath (KS) rice. Table S7. Allele dependency and expression profiles of genes in rice zygotes at 4 HAF. Table S8. Genes in rice zygotes with paternal allele‐dependent expression at 4 HAF. Table S9. Genes in rice zygotes with maternal allele‐dependent expression at 4 HAF. Table S10. Genes in rice zygotes with biallelic expression at 4 HAF. Table S11. Genes with preferential expression from the paternal allele at 4 HAF. Table S12. Genes with preferential expression from the maternal allele at 4 HAF. Table S13. Genes with biallelic expression at 4 HAF. Table S14. Genes with biallelic expression at 18 HAF. Table S15. Genes with preferential expression from the paternal allele at 18 HAF. Table S16. Genes with preferential expression from the maternal allele at 18 HAF. Table S17. Genes with preferential expression from the paternal allele in Modules 13 and 8 at 4 HAF. Table S18. Genes with preferential expression from the maternal allele in Modules 13, 6, and 8 at 4 HAF. Table S19. Genes with biallelic expression in Modules 13, 14, 6, and 8 at 4 HAF. Table S20. GO terms enriched from biallelic expressed genes in Modules 13 at 4 HAF. Table S21. Expression levels of OsBBM1, 2, and 3 in rice zygotes produced by electro‐fusion of gametes isolated from wild type and bbms t [file TPJ-122-0-s001.zip › tpj70305-sup-00023-Table S22.docx]

**Supplementary Table S22. Primers used for PCR**

| Target locus | Forward (5'–3') | Reverse (5'–3') |
| --- | --- | --- |
| Os02g0161900 | TCCGTGGTGGTCAGTA | ACTGCTGTCCCACAGG |
| Os01g0211200 | ATTTCCCAGAACCCGGTCG | AATGGTGGACTCGCTGCTAT |
| Os05g0189300 | CCAATGTTGCCGACGATGAT | GAGTACACTGCGGCTTTACC |
| Os11g0182200 | GAACAGCCTCTTCAACTCCG | TTCTTCGACGTGGGCAGG |
| Os03g0778000 | TGGAGGGAGCACTAGAATGG | TGAAATTTGCTGGTCCTCGC |
| Os04g0321600 | GGACAGCGAAGAAGAGAGGA | TTACAGCCAAGCACACCATG |
| Os01g0153300 | ACAGCAAAAGAACCACCACC | CGGTCCTTCTTCCCCTCG |
| Os03g0279000 | AAGAAGGGGAGGAAGAAGGC | TGATGGTGGGCTTCTTGTTG |
| Os01g0835900 | GCTCGCGGTCAAGAAATCC | TGAGGTTTGCAGCGAATGAC |
| Os10g0418000 | CCAAGGAGTAGGAGAGGCAG | GCCAGCCATTACACAGATTCA |
